# Perineuronal nets and GABAergic cells in the inferior colliculus of guinea pigs

**DOI:** 10.3389/fnana.2013.00053

**Published:** 2014-01-08

**Authors:** Nichole L. Foster, Jeffrey G. Mellott, Brett R. Schofield

**Affiliations:** ^1^School of Biomedical Sciences, Kent State UniversityKent, OH, USA; ^2^Department of Anatomy and Neurobiology, College of Medicine, Northeast Ohio Medical UniversityRootstown, OH, USA

**Keywords:** midbrain, plasticity, auditory, GABA, inhibition, extracellular matrix

## Abstract

Perineuronal nets (PNs) are aggregates of extracellular matrix that have been associated with neuronal plasticity, critical periods, fast-spiking cells and protection from oxidative stress. Although PNs have been reported in the auditory system in several species, there is disagreement about the distribution of PNs within the inferior colliculus (IC), an important auditory hub in the midbrain. Furthermore, PNs in many brain areas are preferentially associated with GABAergic cells, but whether such an association exists in the IC has not been addressed. We used *Wisteria floribunda* agglutinin staining and immunohistochemistry in guinea pigs to examine PNs within the IC. PNs are present in all IC subdivisions and are densest in the central portions of the IC. Throughout the IC, PNs are preferentially associated with GABAergic cells. Not all GABAergic cells are surrounded by PNs, so the presence of PNs can be used to subdivide IC GABAergic cells into “netted” and “non-netted” categories. Finally, PNs in the IC, like those in other brain areas, display molecular heterogeneity that suggests a multitude of functions.

## INTRODUCTION

Perineuronal nets (PNs) are aggregates of extracellular matrix molecules that surround a subset of neurons in many regions of the central nervous system (for review, Celio and Blümcke, 1994; Karetko and Skangiel-Kramska, 2009). PNs appear postnatally and often in association with specific developmental events, for example, in zebra finch song nuclei around the time of song learning (Balmer et al., 2009), or in mouse striatum around the establishment of mature gait (Lee et al., 2008). The main molecular components of PNs are chondroitin sulfate proteoglycans (CSPGs), such as aggrecan and brevican, which are known to inhibit neurite outgrowth (Hockfield et al., 1990; Yamada et al., 1997; Corvetti and Rossi, 2005; Ajmo et al., 2008). These observations have led to the hypothesis that PNs function to reduce structural plasticity following development. This proposal is supported by studies showing renewed plasticity in adults following experimental degradation of PNs (Pizzorusso et al., 2002; Gogolla et al., 2009). More recent work has expanded the list of functions attributed to PNs, including promotion of synaptic plasticity (Bukalo et al., 2001; de Vivo et al., 2013), protection of neurons from oxidative damage (Suttkus et al., 2012; Cabungcal et al., 2013), and binding molecules for transport into the cell (Beurdeley et al., 2012).

Perineuronal nets have been demonstrated in the auditory system at the level of the cochlear nucleus, superior olivary complex, nuclei of the lateral lemniscus, and the inferior colliculus (IC), and are often associated with specific cell types and nuclei (Seeger et al., 1994; Cant and Benson, 2006; Costa et al., 2007; Myers et al., 2012). Descriptions of PNs in the IC vary, with some describing PNs as more numerous in the central nucleus whereas others describe them as more numerous in the surrounding regions (rat: Seeger et al., 1994; Friauf, 2000; dog: Atoji et al., 1997; rhesus: Hilbig et al., 2007). It is unclear whether these differences reflect the species, experimental methods, or some other issue.

Perineuronal nets have been shown in many brain areas to surround GABAergic interneurons preferentially (Kosaka and Heizmann, 1989; Härtig et al., 1992). In many areas, these GABAergic interneurons are further distinguished by immunoreactivity to parvalbumin (Celio, 1986) and by fast physiological properties associated with Kv3.1b subunits in voltage-gated potassium channels (Sekirnjak et al., 1997). It remains to be determined whether PNs in the IC are preferentially associated with GABAergic neurons. This is of interest because many of the GABAergic neurons in the IC project to extrinsic targets, and thus do not fit the definition of local interneuron (González-Hernández et al., 1996; Winer et al., 1996; Peruzzi et al., 1997). GABAergic IC cells have also been classified according to the presence or absence of a perisomatic ring of axosomatic terminals containing vesicular glutamate transporter 2 (VGLUT2; Ito et al., 2009). The relationship of PNs to VGLUT2 perisomatic terminal rings is also unknown.

In this study, we used immunochemistry and staining with *Wisteria floribunda* agglutinin (WFA) to investigate PNs in the IC of guinea pigs, a species widely used in auditory research. The results show that PNs are present throughout the IC and are densest in the central nucleus. In all IC subdivisions, the PNs associate preferentially with GABAergic cells. However, not all GABAergic cells in the IC are surrounded by PNs. The presence or absence of a PN thus provides a marker for distinguishing two groups of GABAergic cells. By comparing PN staining with VGLUT2 staining, we demonstrate that VGLUT2–immunopositive rings are present in guinea pigs and are associated primarily with GABAergic cells that also have PNs. In addition, we describe a serendipitous finding that demonstrates molecular heterogeneity of PNs in the IC. This heterogeneity is consistent with a variety of functions for the PNs.

## MATERIALS AND METHODS

Eleven adult pigmented guinea pigs of either gender were used. Seven were obtained from Elm Hill Labs (Chelmsford, MA, USA) and four were bred at Northeast Ohio Medical University (Rootstown, OH, USA). All procedures were approved by the Institutional Animal Care and Use Committee and administered following the National Institutes of Health guidelines for the care and use of laboratory animals. Efforts were made to minimize suffering and the number of animals used.

Animals were perfused with Tyrode’s solution followed by 250 ml of 4% paraformaldehyde in 0.1 M phosphate buffer (pH 7.6; PB), then 250 ml of 4% paraformaldehyde in 0.1 M PB with 10% sucrose. The brain was removed and stored overnight at 4°C in 4% paraformaldehyde in PB with 25% sucrose. The following day, the cerebellum and cerebral cortex were removed and the brainstem was frozen and cut on a sliding microtome into 30, 40, or 50 μm thick sections. Nine brains were cut in the transverse plane, one in the sagittal plane, and one in the horizontal plane. Tissue from each animal was divided into six series (every sixth section) so that tissue from one animal could be processed in multiple ways.

### TISSUE PROCESSING

Prior to staining, sections were permeablized in 0.2% Triton X-100 in phosphate-buffered saline [0.9% NaCl in 0.01M PB, pH 7.4; phosphate buffered saline(PBS)] for 30 min at room temperature, then blocked in 10% normal goat or donkey serum in 0.2% Triton X-100 and PBS for 1 h, also at room temperature. Sections were then processed for one or more markers as described below.

#### Lectin staining

Perineuronal nets were stained for 1 h with 1% WFA that was conjugated either to AlexaFluor 488 (Vector Labs, Burlingame, CA, USA, product #FL1351) or to biotin (Sigma, product #L1516). Biotinylated WFA was visualized using AlexaFluor 647-conjugated streptavidin (1%; 1 h at room temperature; Invitrogen, Carlsbad, CA, USA).

#### Immunochemistry

All antibodies were applied overnight at 4°C, except when applied concurrently with anti-VGLUT2, which was applied for 48 h at 4°C. All markers were visualized using either an AlexaFluor-conjugated secondary antibody (1:100, Invitrogen) or a biotinylated secondary antibody (1:100, Vector) followed by AlexaFluor-conjugated streptavidin (1:100, Invitrogen).** GABAergic cells were labeled using an antibody to glutamic acid decarboxylase (GAD67; diluted 1:400 or 1:250, Millipore, Billerica, MA, USA, MAB5406). This antibody has been used previously in guinea pig brain (e.g., Xiong et al., 2008; Nakamoto etal., 2013). Cholinergic structures (and PNs) were labeled with an antibody to vesicular acetylcholine transporter (VAChT; 1:50, Santa Cruz Biotechnology, Inc., Santa Cruz, CA, USA, sc7717). The antibody labeled many somata in known cholinergic nuclei of the brainstem (Motts et al., 2008). Controls included primary omission as well as pre-adsorption with VAChT peptide (Santa Cruz, sc7717 P) at 20 times the antibody concentration. Because the antibody produced perisomatic staining in the IC that resembled staining with WFA, a further control was run with pre-adsorption of the VAChT antibody with 500 mM *N*-acetylgalactosamine (GalNac, the target for WFA binding, Vector, product #S-9001) at 25 times the antibody concentration. Glutamatergic terminals were labeled with an antibody to VGLUT2 (diluted 1:2500, Millipore, AB2251). The procedure stained puncta similar in morphology and distribution to glutamatergic IC terminals described previously (Ito et al., 2009). The staining of puncta was eliminated by pre-adsorption with a VGLUT2 control peptide (Millipore, product #AG209). In some cases there was light staining of some IC somata, particularly laterally. This staining was not blocked in the pre-adsorption controls. Moreover, it was present in primary omission controls, indicating binding of the secondary antibody to these cells. Since we used the VGLUT2 antibody to stain terminals, but not somata, we disregarded this cellular staining (Sergeeva and Jansen, 2009). An antibody to neuronal nucleus specific antigen (NeuN) was used as a neuron specific counterstain (diluted 1:500, Millipore, ABN78). Additionally, IC subdivisions were determined by processing one series of sections from each case with an antibody to brain nitric oxide synthase (bNOS; diluted 1:1000, Sigma, N2280; Coote and Rees, 2008; Nakamoto etal., 2013).

Tissue from nine animals was processed for PNs and GAD, from one animal for PNs and VAChT, from three animals for VAChT and GAD, and from two animals for PNs, GAD, and VGLUT2. Following staining, sections were mounted on gelatin-coated slides, air-dried, and coverslipped with DPX mountant (Sigma-Aldrich, St. Louis, MO, USA).

### DATA ANALYSIS

Quantitative analyses were performed using a Neurolucida system (MBF Bioscience) attached to a Zeiss AxioImager Z2 fluorescence microscope (Carl Zeiss MicroImaging, Inc., Thornwood, NY, USA) or to a Zeiss Axioplan II microscope (Zeiss). Individual cells, nets, and VGLUT2 rings were plotted within the IC. Cells that were associated with two or more labels were plotted with different symbols that denoted the specific combination of markers. Numerical summaries of the markers were used for quantitative analyses of double and triple-labeling. IC subdivisions were identified according to Coote and Rees (2008) by aligning the tracing with a nearby section processed for bNOS. Plots were created using Neurolucida (MBF Bioscience, Williston, VT, USA) and refined using Adobe Illustrator (Adobe Systems, Inc., San Jose, CA, USA). Density of PNs was calculated by finding the area of each subdivision of each section using Neurolucida and multiplying by section thickness to obtain the volume in which the stained nets were counted (section thickness was estimated as the thickness setting on the microtome during sectioning). The values across all three axes were not corrected for tissue shrinkage due to fixation and processing. The total number of PNs in the subdivision was divided by this “section volume” to find density.

Analysis of tissue containing staining for VGLUT2 was complicated because penetration of the VGLUT2 staining was incomplete, i.e., staining was not present through the entire depth of the tissue. To correct for this lack of staining in the deeper parts of the sections, quantitative analysis of the two cases stained for PNs, GAD67, and VGLUT2 was restricted to portions of the section that showed clear VGLUT2 staining. This was accomplished by plotting the data with a 63X objective (NA = 1.4), taking care to focus carefully on each labeled structure. The plotted symbol thus carried a precise Z (depth) coordinate that could be used to filter the markers according to the depth of each structure in the section. The depth of penetration of the VGLUT2 labeling was assessed separately for each subdivision of each section, and all markers outside of this depth range were excluded from further analyses by exporting the marker coordinates from Neurolucida to Microsoft Excel and sorting them based on the Z coordinate. The area lacking VGLUT2 stain ranged from 4 to 13 μm thick, with an average of 9 μm of tissue excluded from analysis.

Numerical analyses and graphs were completed with Microsoft Excel. All numerical results are reported as mean ± SEM, unless otherwise noted. Photomicrographs were taken using a Zeiss Imager Z1 fluorescence microscope with an AxioCam HRm camera (Zeiss). Addition of scale bars and arrows, cropping, pseudocoloring, and adjustment of levels were done in Adobe Photoshop (Adobe Systems).

## RESULTS

### CHARACTERISTICS OF IC PNs: DISTRIBUTION, ASSOCIATION WITH GABAERGIC CELLS, AND MOLECULAR HETEROGENEITY

#### WFA-labeled PNs are most dense in central portions of the IC

**FIGURE 1:**
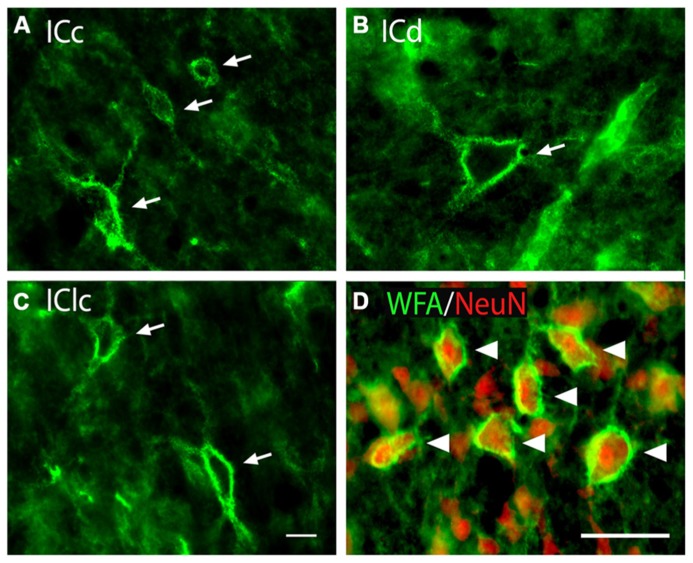
**Photomicrographs of PNs (arrows) in the IC (A–C)**. WFA-labeled PNs in each IC subdivision showing similarity of nets across subdivisions as well as variation in staining intensity, size, and morphology. GP696. Scale bar = 20 μm. **(D)** WFA and anti-NeuN staining in the ICc. Arrowheads indicate WFA-labeled PNs (green) around neurons stained with the neuron-specific marker NeuN (red). ICc, ICd, IClc: inferior colliculus central nucleus, dorsal cortex, and lateral cortex, respectively. GP710. Scale bar = 50 μm.

*Wisteria floribunda* agglutinin-labeled PNs are readily identifiable in the IC as extracellular aggregates of WFA staining around certain neurons. PNs with a similar range of morphologies are present across the major IC subdivisions (**Figures 1A–C**). PNs vary in staining intensity and size (**Figure 1A**). PNs also vary in shape, presumably reflecting the morphology of the neurons that they surround (e.g., compare small round net, upper arrow in **Figure 1A**, with highly elongated net in **Figure 1C**, lower arrow). WFA-labeled PNs in the IC commonly cover somatic extensions (**Figure 1B**), described in other studies as proximal dendrites (Celio and Blümcke, 1994). We confirmed that IC PNs surround neurons by staining with anti-NeuN, a neuronal marker (**Figure 1D**). Overall, about 90% of WFA-labeled PNs in IC surround NeuN-positive cells (the remaining NeuN-negative cells may or may not be neurons, as certain neuron types have been found to be NeuN-negative; Mullen et al., 1992).

**FIGURE 2:**
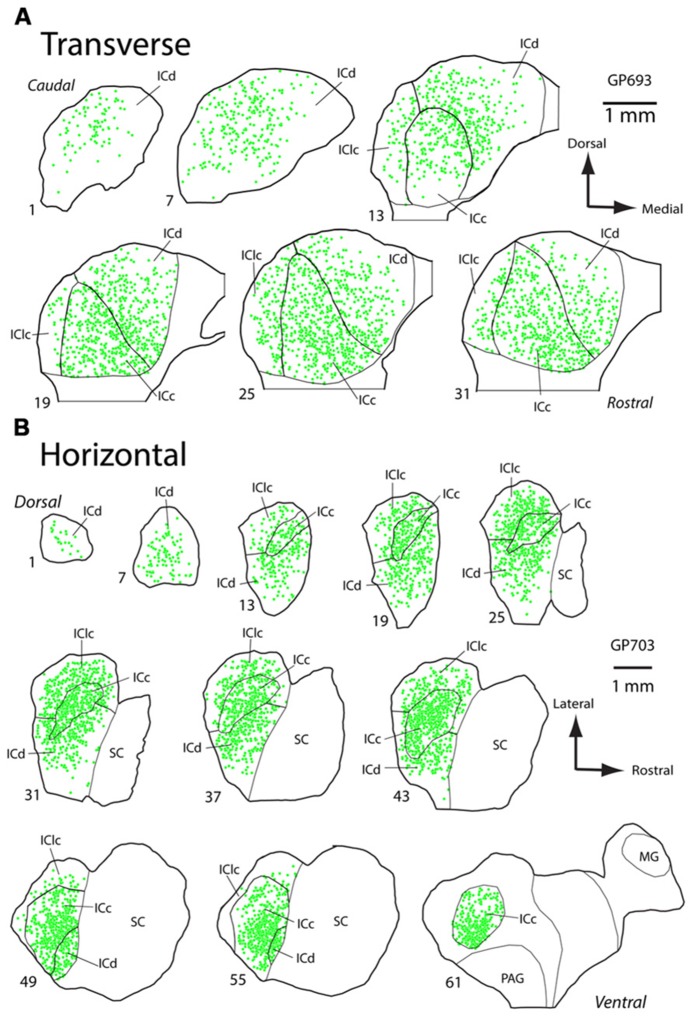
**Plots of WFA-labeled PNs in the inferior colliculus (IC).** **(A)** Plot showing the distribution of WFA-labeled PNs (green circles) in transverse sections through the IC. Sections progress from caudal to rostral. Medial is right, dorsal is up. Scale bar = 1 mm. Case GP693. **(B)** Plot showing distribution of WFA-labeled PNs (green circles) in horizontal sections through the IC. Sections progress from dorsal to ventral. Rostral is right, lateral is up. Scale bar = 1 mm. Case GP703.

**FIGURE 3:**
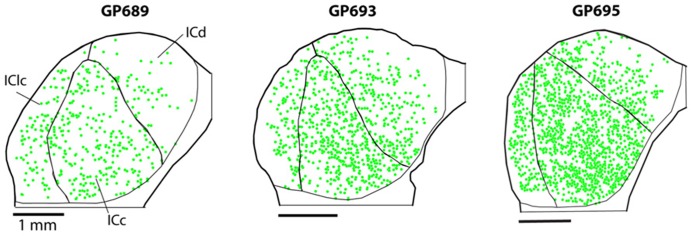
**Plots of WFA-labeled PNs (green circles) showing the variation in PN density across three cases.** In each case, the average PN density is highest in the ICc, but the absolute density of PNs varies, ranging from low density in case GP689, to intermediate level in case GP693, and high density in case GP695. Transverse plane; medial is right, dorsal is up. Scale bars = 1 mm.

**FIGURE 4:**
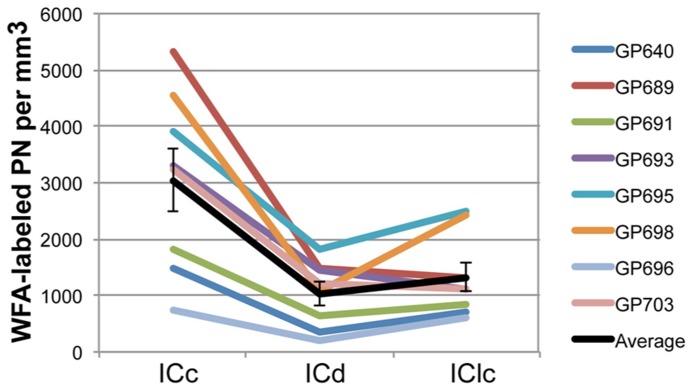
**Graph of PN density in each IC subdivision in eight animals.** The values across subdivisions are connected for each individual case to facilitate comparisons between the cases; note that in all cases the ICc has the highest density of PNs. Black line indicates the average values and SEM. On average, ICc contains a higher density of WFA-labeled PNs than ICd or IClc. Error bars = SEM.

PNs are quite numerous in the IC, with an average density of 1,758 ± 329 PNs per mm^3^. However, the density of PNs is not uniform across the IC. **Figure 2** shows plots of WFA-labeled PNs (green circles) in representative cases in the transverse and horizontal planes. PNs tend to be more dense centrally and less dense near the outer margins of the IC, as seen clearly in the transverse plane (**Figure 2A**). The low density of PNs dorsally and at medial, lateral, and caudal extremes is also readily seen in horizontal sections (**Figure 2B**). An area of highest PN density is located in ventral central nucleus of the inferior colliculus (ICc) and spreads into the dorsal cortex of the inferior colliculus (ICd; e.g., **Figure 2B**, sections 49, 55). While the general patterns of staining were consistent across cases (e.g., the density was always highest in the ICc), the absolute density varied between cases. **Figure 3** shows representative transverse plots of PNs (green circles) in roughly equivalent sections through the middle of the IC in three cases. Visual inspection shows a range from lowest density (left-most plot) to the highest density (right-most plot). Despite the variation across cases, the relative density of PNs was always highest in the central nucleus (**Figure 4**). Examination of values from individual cases shows, for example, that in case GP640 the density is highest in the ICc, but this value is lower than that in any IC subdivision in case GP695.

#### Most WFA-labeled PNs surround GABAergic IC cells

**FIGURE 5:**
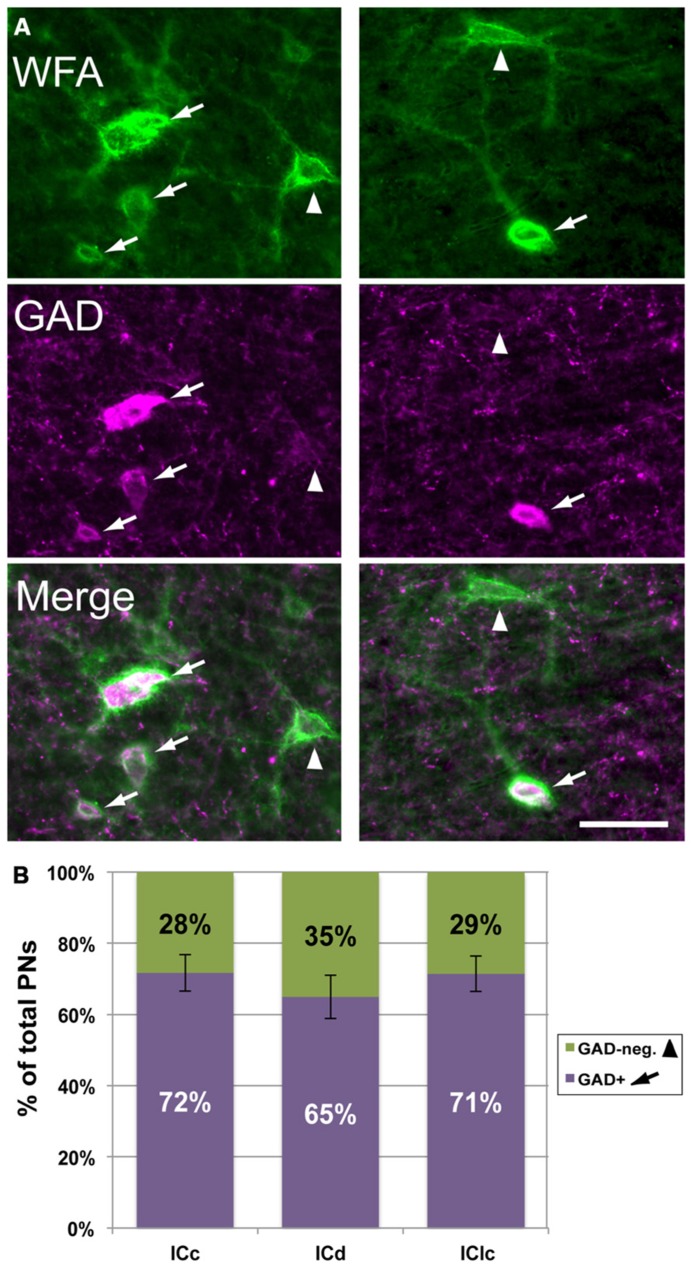
**WFA-labeled PNs surround both GAD+ and GAD-negative IC cells.** **(A)** Photomicrographs from IClc (left column) and ICd (right column) showing WFA-labeled PNs (top row) and cells stained with antibodies to GAD (middle row). Bottom row shows the merged images, revealing PNs around GAD+ (arrows) and GAD-negative (arrowheads) IC cells. Scale bar = 50 μm. Case GP691. **(B)** Graph showing the percentages of WFA-labeled PNs that surround GAD+ (purple bars) vs. PNs that surround GAD-negative (green bars) cells in each IC subdivision. The symbols in the legend correspond to those in **(A)**. Error bars = SEM; data are averaged across 74 sections in nine animals.

As described in the Introduction, in many brain areas PNs are preferentially associated with GABAergic cells. We examined this issue for the IC by staining nets with WFA and presumptive GABAergic cells with an antibody to glutamic acid decarboxylase (GAD), a specific marker of GABAergic neurons. **Figure 5A** shows that WFA-labeled PNs in the IC surround GAD-immunopositive cells (“GAD+”; arrows) and GAD-negative cells (arrowheads). On average, a majority (70 ± 4%) of WFA-labeled PNs surround GAD+ cells. This is true for each IC subdivision individually (**Figure 5B**). While a minority of WFA-labeled PNs surround GAD-negative cells, this still corresponds to about 30% of the total WFA-labeled PN population in IC.

#### An antibody to VAChT protein stains IC PNs

**FIGURE 6:**
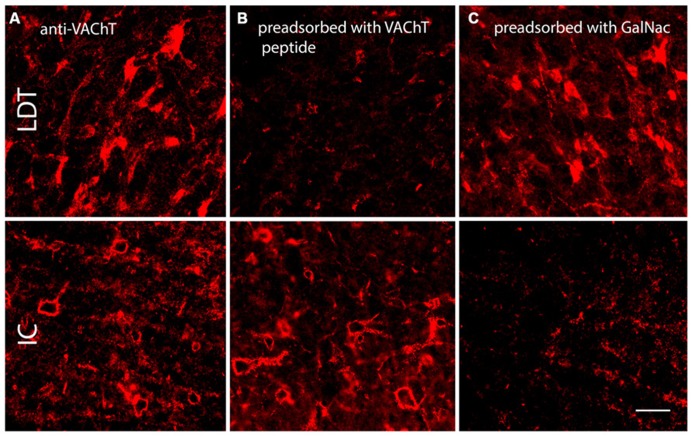
**Photomicrographs showing anti-VAChT staining in the laterodorsal tegmental nucleus (LDT, a midbrain cholinergic nucleus) and the IC under different pre-adsorption conditions.** **(A)**. Somatic staining in the LDT (top row) and PN-like staining in the IC (bottom row) following application of anti-VAChT antibody to guinea pig tissue. **(B)**. Following pre-adsorption of the anti-VAChT antibody with VAChT protein, somatic staining in the LDT is abolished (top) but PN-like staining in the ICc remains (bottom). **(C)**. Following pre-adsorption of the anti-VAChT antibody with *N*-acetylgalactosamine (GalNac, the target of WFA binding), somatic staining is present in the LDT (top), but net-like staining in the ICc is abolished (bottom). Scale bar = 50 μm. Case GP691.

Antibodies to VAChT are commonly used to mark cholinergic neurons and synaptic terminals (e.g., Gilmor et al., 1996). We initially applied this antibody to guinea pig tissue with the intent of labeling cholinergic boutons in the IC. The IC contains few or no cholinergic cell bodies, but we expected to see anti-VAChT-labeled cholinergic cells in numerous other brainstem nuclei (as revealed by staining with another cholinergic marker, choline acetyltransferase; Motts et al., 2008). Initial trials with VAChT immunostaining produced somatic staining in known cholinergic nuclei [e.g., laterodorsal tegmental nucleus (LDT), a midbrain cholinergic center] but did not label any structures in the IC. When we increased the concentration of primary antibody (in attempts to label the cholinergic boutons), the result was PN-like staining in the IC, in addition to the somatic staining in LDT and elsewhere (**Figure 6A**), but no staining of boutons in the IC. When we treated the VAChT antibody with VAChT peptide before application to the tissue (i.e., in a pre-adsorption control), the somatic staining in LDT was abolished, but net-like staining in IC was still present, indicating that the net-like staining is unlikely to be the result of binding to the VAChT protein (**Figure 6B**). When the VAChT antibody was pre-adsorbed with GalNac (the target for WFA binding; Spicer and Schulte, 1992), somatic staining in LDT was present, but the net-like staining in IC was abolished (**Figure 6C**). These results suggest that the net-like staining by anti-VAChT is not related to cholinergic transmission.

#### WFA and α-VAChT-labeled PNs share many characteristics

**FIGURE 7:**
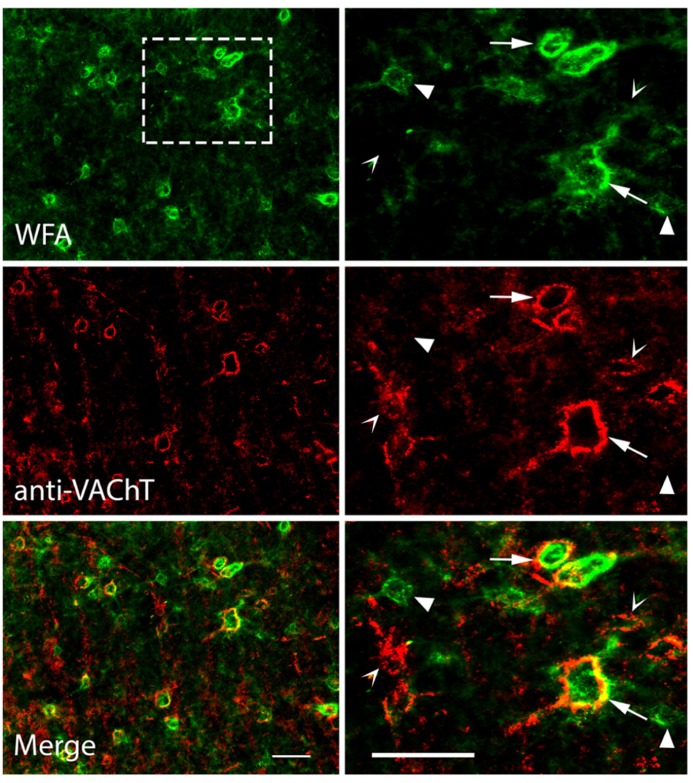
**Photomicrographs showing anti-VAChT co-staining with WFA-labeled PNs in the IC.** Top row: PNs in the ICc are stained with WFA. Right column is an enlargement of boxed area in top left photograph. Middle row: the same areas as top row, viewed to reveal anti-VAChT staining. Bottom row: merged images; yellow color indicates regions of direct overlap of green and red signals. Solid arrowheads: PNs labeled with WFA only; Open arrowheads: PNs labeled with VAChT only. Arrows: PNs labeled with both WFA and VAChT. Scale bars = 50 μm. Case GP691.

To our knowledge, anti-VAChT has never been used as a marker for PNs, so we applied WFA and anti-VAChT to the same tissue to examine the relationships between the two types of staining. **Figure 7** shows the results of differential staining with these two markers in the IC. Many PNs stain with both markers. WFA-labeled PNs are stained with anti-VAChT 49% of the time, while VAChT-labeled PNs are stained with WFA 84% of the time. Thus, some nets stain with both markers (**Figure 7**, arrows), some stain only with WFA (**Figure 7**, solid arrowhead) and the smallest population stains only with anti-VAChT (**Figure 7**, open arrowhead). The patterns of single and double staining suggest molecular heterogeneity of the nets in the IC; such heterogeneity is a common feature of nets in many brain areas (Ajmo et al., 2008; Blosa et al., 2013).

**FIGURE 8:**
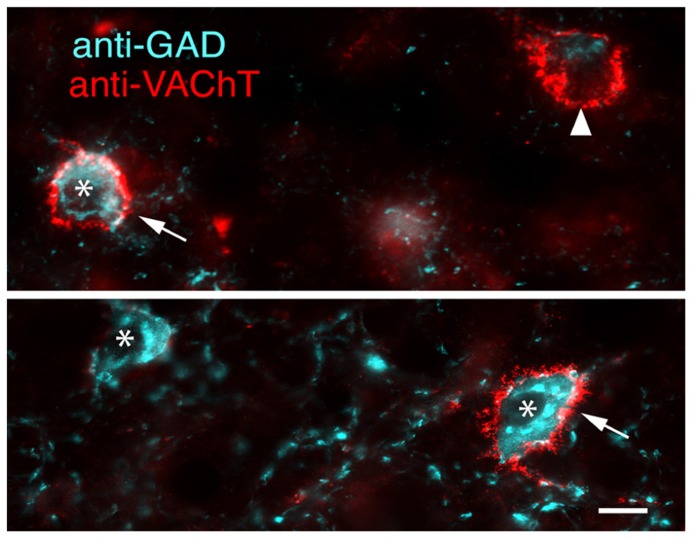
**Merged photomicrographs showing anti-VAChT labeled PNs surrounding either GAD+ cells (arrows) or a GAD-negative cell (arrowhead).** A GAD+ cell without an anti-VAChT labeled PN is also present (asterisk). Images from ICc. Scale bar = 10 μm. Case GP613.

The high degree of co-staining of PNs with WFA and anti-VAChT ensure similar distributions and morphologic properties. PNs labeled with anti-VAChT have an average overall density of 1,699 ± 431 PN per mm^3^. Density of VAChT-labeled PNs is consistently higher in ICc (2,987 ± 843 PN per mm^3^) than in ICd (1,209 ± 410 PN per mm^3^) or lateral cortex of the inferior colliculus (IClc; 522 ± 118 PN per mm^3^). PNs stained by anti-VAChT are also similar to WFA-stained PNS in their association with GAD+ cells (**Figure 8**, arrows) and GAD-negative cells (**Figure 8**, arrowhead). The majority of anti-VAChT-labeled PNs (69 ± 5%) are associated with GAD+ cells. This is also true in each IC subdivision: 70 ± 16% in ICc, 67 ± 7% in ICd, and 64 ± 9% in IClc.

### USING PNs TO SUBDIVIDE IC GABAergic CELLS

**FIGURE 9:**
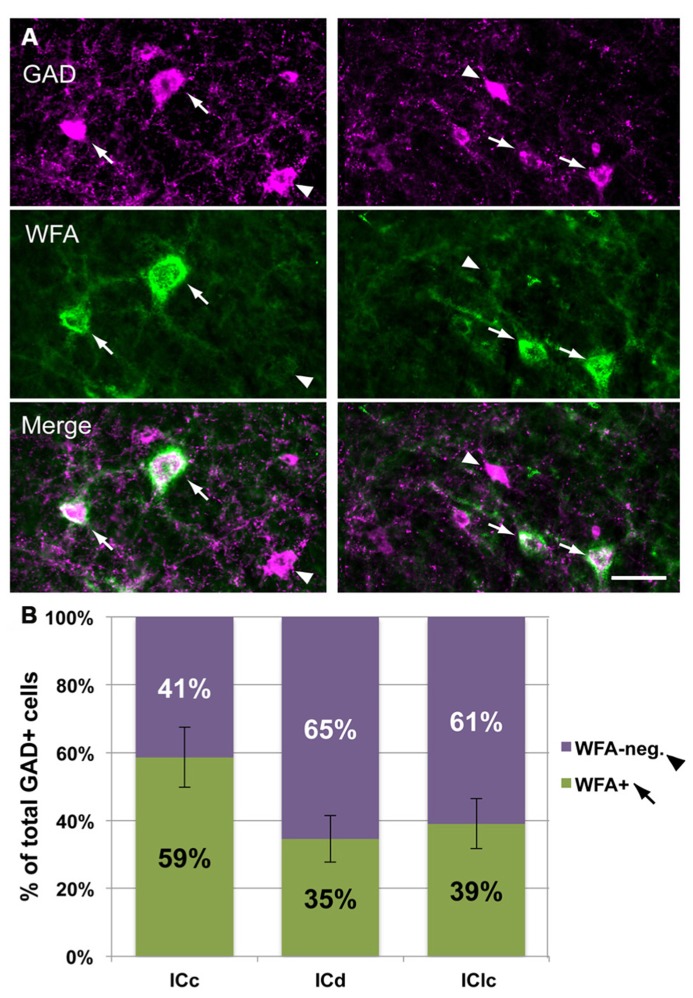
**A subset of IC GAD+ cells are surrounded by WFA-labeled PNs.** **(A)**. Photomicrographs from ICc (left column) and IClc (right column) showing GAD+ cells that are surrounded by PNs (arrows) and GAD+ cells that are *not* surrounded by PNs (arrowheads). Top row: two different areas stained for GAD. Middle row: same areas as top row, showing WFA staining for PNs. Bottom row: merged image showing GAD+ cells with or without PNs. Scale bar = 50 μm. Case GP691. **(B)**. Graph showing the percentage of GAD+ cells in each IC subdivision that are surrounded by PNs (green bars) or not surrounded by PNs (purple bars). Error bars = SEM; data were averaged across 74 sections in nine animals.

The results presented thus far have focused on the PNs and whether or not they surround GAD+ cells. The tissue used for these analyses also revealed that many GAD+ cells in the IC are *not* surrounded by nets (**Figure 9**). In the IC overall, 44 ± 7% of GAD+ cells were surrounded by a WFA-labeled PN. In the ICc, a majority (59 ± 9%) of GAD+ cells are netted (**Figure 9B**). The opposite pattern is observed in the ICd and IClc, where PNs surround a minority of the GAD+ cells (**Figure 9B**). Thus, GABAergic IC cells can be divided into PN-surrounded (“netted”) and non-PN-surrounded (“non-netted”) categories.

**FIGURE 10:**
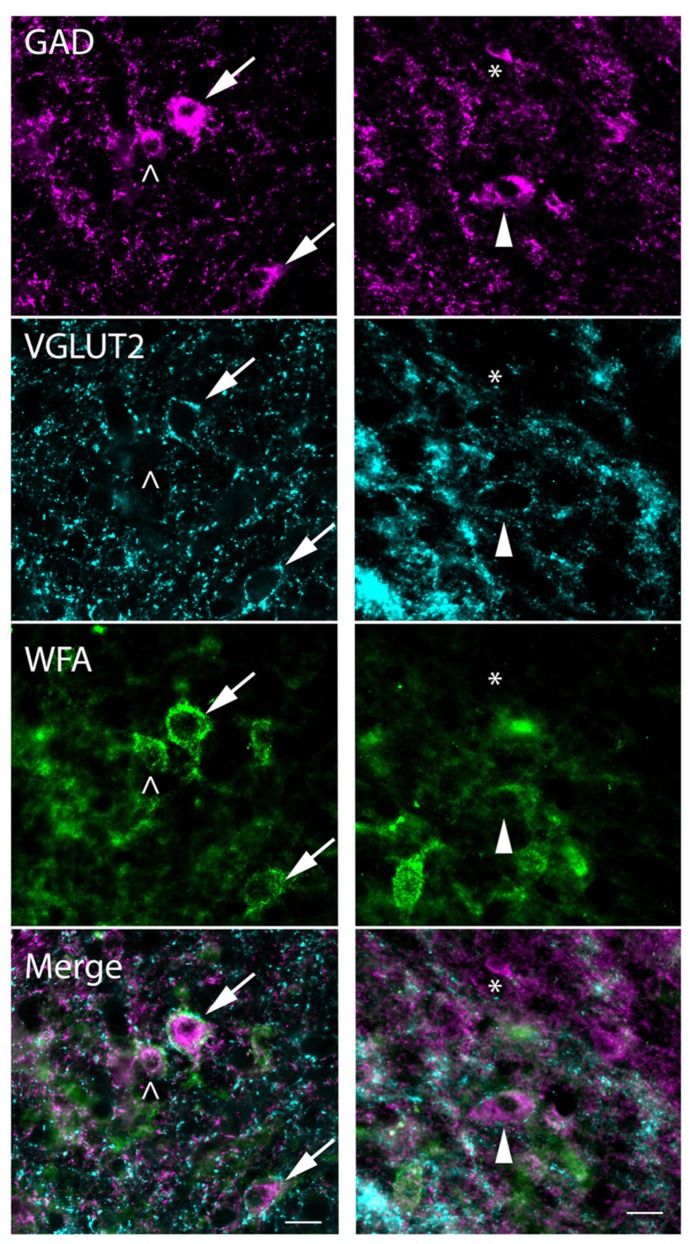
**Photomicrographs showing association of PNs and VGLUT2-immunopositive perisomatic bouton rings with GAD+ cells.** Examples are shown from the ICd (left column) and ICc (right column). Each column shows a region stained for GAD (top row), VGLUT2 (second row), and WFA (third row). Bottom row: merged images showing all three markers. GAD cells, top row, are indicated by four different symbols that represent the four possible relationships with PNs and VGLUT2 rings. Arrows (left column) show GAD+ cells that have VGLUT2+ rings and WFA-stained PNs. Open arrowhead (left column) shows a GAD+ cell that lacks a VGLUT+ ring but is surrounded by a PN. Solid arrowhead (right column) shows a GAD+ cell that is surrounded by a VGLUT2 ring but lacks a WFA-stained PN. Asterisk (right column) is adjacent to a small GAD+ cell that has neither a PN nor a VGLUT2 ring. Note that one PN (bottom left corner of the right column) surrounds a GABA-negative cell. Scale bar = 20 μm. Case GP710.

The presence of distinct perisomatic rings of glutamatergic terminals that are immunopositive for VGLUT2 (VGLUT2+) has been used to distinguish subsets of GAD+ cells in the IC of rats and mice (Ito et al., 2011). We report here that VGLUT2+ terminal rings also surround a subset of GAD+ cells in guinea pigs, and that this population overlaps primarily with PN-surrounded GAD+ cells. **Figure 10** shows the four different patterns of labeling associated with GAD+ cells. Some GAD+ cells are surrounded by distinctive perisomatic rings of VGLUT2+ boutons as well as WFA-stained PNs (**Figure 10**, arrows). Other GAD+ cells lack the VGLUT2 ring but do have a PN (**Figure 10**, left column, open arrowhead). The third type of GAD+ cell has a VGLUT2 ring but lacks a PN (**Figure 10**, right column, arrowhead). Finally, the fourth staining pattern is a GAD+ cell that has neither VGLUT2 ring or PN (cell adjacent to asterisk in **Figure 10**, right column). The four staining patterns were present in very different numbers. The majority (88 ± 1%) of GAD+ cells with VGLUT2 rings also were surrounded by PNs. Even so, the rings were associated with only a portion of the netted cells (average 28 ± 15% across all IC subdivisions). GAD+ cells without nets were almost always lacking VGLUT2 rings; the exceptions (e.g., **Figure 10**, arrowhead) were rare (averaging 2 ± 0.2% of the non-netted GAD+ cells). A substantial proportion of IC GAD+ cells, then, lack both PNs and VGLUT2 rings.

## DISCUSSION

We found that PNs are present in all IC subdivisions in guinea pigs, but are most concentrated in central regions of IC. This represents a concentration in the ICc as well as large numbers of nets in the adjacent parts of surrounding subdivisions. PNs also display molecular heterogeneity as reflected by differential staining with two different markers: WFA and anti-VAChT. In all IC subdivisions, PNs are preferentially associated with GABAergic cells. We also demonstrated that IC GABAergic cells can be subdivided into PN-surrounded (“netted”) and non-netted categories. In the ICc, netted and non-netted GAD+ cells are about equally abundant. In the other IC subdivisions, the majority of GAD+ cells lack PNs. Lastly, we identified perisomatic rings of VGLUT2+ glutamatergic terminals that surround a relatively small percentage of IC GAD+ cells. Most of the cells with these VGLUT2 rings were also surrounded by PNs.

### TECHNICAL CONSIDERATIONS

*Wisteria floribunda* agglutinin is widely used as a marker for PNs across numerous species (human: Belichenko et al., 1999; rat, monkey: Härtig et al., 1999; bison: Härtig et al., 2001; gerbil: Cant and Benson, 2006; mouse: Cabungcal et al., 2013). It is generally accepted that WFA stains a majority of PNs, but not all types. Ajmo et al. (2008) found that staining with WFA and an antibody to the CSPG brevican identified some PNs that were brevican-reactive, some PNs that were WFA-reactive, and some PNs that were reactive for both markers. In cerebral cortex and hippocampus, the majority of PNs were reactive for only brevican (Ajmo et al., 2008). More recently, Lendvai et al. (2012) report that in human hippocampus, an anti-brevican antibody labels few PNs, but many axonal coats (aggregates of ECM around individual synapses). Further, Blosa et al. (2013) found that PNs in the medial nucleus of the trapezoid body (a brainstem auditory nucleus) contain both aggrecan and brevican, and that PNs labeled with an antibody to aggrecan were also generally labeled with WFA. This evidence, along with the previous use of WFA as a PN label in IC (Seeger et al., 1994; Costa et al., 2007; Hilbig et al., 2007) leads us to conclude that WFA is staining many PNs in the guinea pig IC.

We are not aware of any previous observation that anti-VAChT antibody can label PNs, as it is usually a marker for cholinergic terminals (e.g., Gilmor et al., 1996). It is important to note that PNs were stained with this antibody only when we used concentrations of primary antibody higher than that needed to stain cholinergic cell bodies. Pre-treating the antibody with VAChT protein and then applying it to the tissue at these high concentrations abolishes cellular staining in LDT, a known cholinergic nucleus, but not the PN-like staining in IC. Further, pre-treating with GalNac (the target for WFA binding) abolishes PN-like staining in the IC, but not the cellular staining in cholinergic nuclei. We also found a high level of co-staining between WFA and anti-VAChT markers; the majority of anti-VAChT-stained structures were also stained with WFA. These findings led us to conclude that the anti-VAChT antibody stains PNs in IC (note: additional PNs were stained with both WFA and VAChT in other brain areas in our experiments; we did not analyze these results, but they suggest that the VAChT-staining variety of nets is not limited to the IC). An interesting and unexpected finding was that a small minority of IC PNs stains with anti-VAChT antibody but not WFA. This led us to the conclusion that anti-VAChT and WFA probably do not share a single binding site. It is possible that anti-VAChT binds to a specific CSPG (brevican, for example) or binds based on the CSPG sulfation pattern, as multiple patterns of sulfation can be present in PNs of a single nucleus (Blosa et al., 2013). Either of these possibilities would have functional implications. According to Yamaguchi (2000), expression of different types of CSPGs could affect the structure of the PN, and determine the level at which it discourages neurite growth. Different sulfation patterns of CSPGs can affect the PN’s affinity for binding extracellular calcium (Vigetti et al., 2008), which has many functional implications for the PN-surrounded cell. Future experiments may show additional heterogeneity in the PNs of the IC; understanding the heterogeneity may prove essential to understanding the functions of the nets.

In this study, we interpret GAD-negative cells as non-GABAergic, an interpretation that relies on successful GAD immunostaining. Our conclusions are based on interpreting GAD-negative cells at the same depth at which GAD-positive cells were observed (often in close proximity to the immunonegative cells). Nonetheless, we cannot rule out a small number of false negatives. Beyond this, we conclude that GAD-negative cells in the IC are almost certainly glutamatergic. There are no IC neurons that express glycine (Merchán et al., 2005) and few or none that express acetylcholine, serotonin, dopamine, adrenaline or noradrenaline (Klepper and Herbert, 1991; Tong et al., 2005; Motts et al., 2008). However, many IC cells express VGLUT2, identifying them as glutamatergic (Ito et al., 2011), and in a subsequent report Ito and Oliver (2012) considered all IC cells to be GABAergic or glutamatergic. Based on this evidence, we use the terms GAD-negative, non-GABAergic, and glutamatergic interchangeably in this discussion, with the understanding that a few GAD-negative cells may actually express a neuromodulator.

### PN DISTRIBUTION IN IC

We have reported here a non-uniform distribution of PNs in guinea pig IC. When subdivisions are identified, ICc consistently shows the highest density of PNs. However, PNs are not confined to any one IC subdivision in guinea pigs, nor are they distributed evenly within subdivisions. We noted a higher density of PNs in the ventral ICc as compared to dorsal ICc, suggesting that PNs might be more numerous in regions responding to high frequencies. Such an organization might reflect the association of PNs with cells that can fire at higher rates (Hilbig et al., 2007), but we could not discern a regular gradient that appeared to correlate with the overall tonotopic map in the ICc. The density of PNs also varied in the other subdivisions, usually being lowest near the outer surfaces of the IC and highest near the borders with the ICc, but once again there were variations between animals that make more general statements of a “typical” pattern hard to identify. Studies of PN distribution in the IC of other species agree that the density is non-uniform but disagree about areas of high versus low density of PNs. Studies in rats describe more numerous PNs in cortical regions (Seeger et al., 1994; Bertolotto et al., 1996) or in central IC (Friauf, 2000). Hilbig et al. (2007) describe more numerous and more heavily stained PNs in cortical IC (vs. central IC) in rhesus monkey as well. Atoji et al. (1997) described sparse PNs around the periphery of ICc in dogs. Our result of more numerous PNs in central regions of IC is not identical to any of the previous reports, even those describing PNs in central IC, such as Atoji et al. (1997). Few of the studies cited above focus on PNs in the IC (some are on PNs in the auditory brainstem, while others are broader surveys of PN staining across many brain areas). It remains to be determined how much of the apparent variability is due to species differences, technical differences, or perhaps to changes in PN expression with age.

### POSSIBLE FUNCTIONAL IMPLICATIONS

It has been suggested that PNs affect cellular function in numerous ways, including reducing structural plasticity (Corvetti and Rossi, 2005), promotion of synaptic plasticity (Bukalo et al., 2001; de Vivo et al., 2013), binding molecules for cellular uptake (Beurdeley et al., 2012), and protecting neurons from oxidative stress (Suttkus et al., 2012). Additionally, Vigetti et al. (2008) reported that the sulfation patterns of CSPGs (the main component of PNs) can affect the opening of voltage-gated channels via their ability to bind extracellular calcium. This finding implies that the presence of a PN could also affect the excitability of a neuron. In the IC, understanding how PNs affect cellular function relies on understanding which neurons are PN-surrounded.

In a study of the rhesus monkey, Hilbig et al. (2007) described a close regional association throughout the auditory pathway between PNs and staining for the Kv3.1b protein, an isoform of the Kv3.1 potassium channel implicated in high frequency neural firing (Rudy et al., 1999; Macica et al., 2003). According to Weiser et al. (1995), staining for Kv3.1b channels in rat is heavier in cortical regions of IC than in the ICc, which matches the described distribution of PNs in the rat (Seeger et al., 1994; Bertolotto et al., 1996). PNs surround fast-spiking GABAergic interneurons in cerebral cortex, and have been demonstrated to protect them from oxidative stress *in vivo* (Cabungcal et al., 2013). Based on these studies, PN-surrounded cells in IC may be fast-spiking and highly metabolically active.

The discussion here, as for many discussions of PNs, has focused on inhibitory cells, and particularly on GABAergic cells. Our results show that some PNs in the IC surround GAD-negative cells, most or all of which are likely to be glutamatergic. In fact, PNs have been identified around glutamatergic cells in other brain areas, including pyramidal cells in neocortex (Hausen et al., 1996). Glutamatergic cells make up about 75% of IC cells in guinea pigs (unpublished observations). Combining this observation with the fact that PNs surround fewer GAD-negative than GAD+ cells suggests that PN surround a relatively small portion of the glutamatergic IC population. It remains to be determined (in the IC or elsewhere) whether PNs serve similar functions for inhibitory and excitatory cells. It will be of interest in future studies to try to identify these functions and perhaps discover what, if anything, differentiates the cells that have nets from those that do not.

Another way to characterize PN-surrounded IC cells is by their projections. In many brain areas, the PNs have been associated with GABAergic interneurons. We have shown a close association of PNs with GABAergic cells in the IC. However, it is not clear whether any of these GABAergic neurons are interneurons; rather, IC GABAergic cells are known for their long-range projections to the medial geniculate (MG) body and to the contralateral IC (Winer et al., 1996; González-Hernández et al., 1996; Peruzzi et al., 1997). These projecting cells probably also have a number of local collaterals, and thus contribute to local circuits as well as extrinsic projections (Oliver et al., 1991). We have reported preliminary data showing that PNs are associated with at least some of the GABAergic IC cells that project to the auditory thalamus (Foster et al., 2013). Additional experiments will be required to determine the extent to which PNs are associated with each of the various targets of IC projections.

## CONCLUSION

We conclude that guinea pigs display a non-uniform distribution of PNs in the IC, with PNs being more numerous in the central nucleus than in surrounding regions. IC PNs preferentially associate with GABAergic cells and are molecularly heterogeneous, consistent with descriptions of PNs in other species and brain areas. Further, IC PNs can be used to subdivide GABAergic IC cells into netted and non-netted populations. Further study of PN-surrounded IC cells is needed to understand the functional implications of the presence of these structures.

## AUTHOR CONTRIBUTIONS

Nichole L. Foster, Brett R. Schofield: designed research and wrote the paper; Nichole L. Foster, Jeffrey G. Mellott, and Brett R. Schofield: performed research and analyzed data.

## Conflict of Interest Statement

The authors declare that the research was conducted in the absence of any commercial or financial relationships that could be construed as a potential conflict of interest.

## ABBREVIATIONS

bNOS: brain nitric oxide synthase
CSPG: chondroitin sulfate proteoglycan
GAD: glutamic acid decarboxylase
GalNac: *N*-acetylgalactosamine
IC: inferior colliculus
ICc: central nucleus of the inferior colliculus
ICd: dorsal cortex of the inferior colliculus
IClc: lateral cortex of the inferior colliculus
LDT: ilaterodorsal tegmental nucleus
MG: medial geniculate body
NeuN: neuronal nucleus specific antigen
PAG: periaqueductal gray
PBS: phosphate buffered saline
PN: perineuronal net
SC: superior colliculus
VAChT: vesicular acetylcholine transporter
VGLUT2: vesicular glutamate transporter 2
WFA: *Wisteria floribunda* agglutinin
IC: inferior colliculus
